# Behavioral Characterizing of CD24 Knockout Mouse—Cognitive and Emotional Alternations

**DOI:** 10.3390/jpm11020105

**Published:** 2021-02-06

**Authors:** Keren Nitzan, Roni Toledano, Shiran Shapira, Nadir Arber, Ravid Doron

**Affiliations:** 1 Department of Education and Psychology, The Open University Israel, Rannana 4353701, Israel; kfridel@gmail.com (K.N.); roni.zara@gmail.com (R.T.); 2 The Integrated Cancer Prevention Center, Tel Aviv Sourasky Medical Center, Tel Aviv 6423906, Israel; shiranshapira@gmail.com (S.S.); nadira@tlvmc.gov.il (N.A.); 3 Department of Molecular Genetics and Biochemistry, Sackler Faculty of Medicine, Tel Aviv University, Tel Aviv 69978, Israel

**Keywords:** CD24, cognition, anxiety, depression, behavior

## Abstract

CD24 is a small, glycophosphatidylinositol-anchored cell surface protein, mostly investigated with respect to cancer, inflammation, and autoimmune diseases. CD24 knockdown or inhibition has been used to test various biochemical mechanisms and neurological conditions; however, the association between CD24 and behavioral phenotypes has not yet been examined. This study aims to characterize cognitive and emotional functions of CD24 knockout mice (CD24^−/−^ )compared with CD24 wild-type mice at three time-points: adolescence, young adulthood, and adulthood. Our results show that CD24^−/−^ mice exhibited better cognitive performance and less anxiety-like behavior compared with WT mice, with no effect on depression-like behavior. This phenotype was constant from childhood (2 months old) to adulthood (6 months old). The results from our study suggest that CD24 may influence important behavioral aspects at the whole-organism level, which should be taken into consideration when using CD24 knockout models.

## 1. Introduction

CD24 is a small, glycophosphatidylinositol-anchored cell surface protein. First recognized for its role in cell adhesion [1] and differentiation [2], it was soon discovered to have a diverse array of functions. CD24 functions were mostly investigated concerning cancer [3]. CD24 is overexpressed in various human malignancies (cervical, gliomas, breast, pancreas, esophageal squamous cell carcinoma, gastric, bladder, and colorectal). In in-vivo and in-vitro cancer models, it was suggested that CD24 promotes colorectal, cervical, and breast cancer cell proliferation [4,5,6,7]. CD24 is also considered a marker for cancer [8] and thus was proposed as a diagnostic tool for cancer [9]. CD24 may also be involved in adaptive immunity, inflammation, and autoimmune diseases and in the central nervous system (CNS). Nevertheless, its behavioral characterization has never been examined.

In the immune system, CD24 is expressed on B cells, T cells, neutrophils, and macrophages, among other hematopoietic cells [10]. CD24 is thought to be involved in various autoimmune diseases. In a multiple sclerosis (MS) mouse model (experimental autoimmune encephalomyelitis; EAE), CD24 expression on CNS resident lymphocytes facilitated disease development and increased the severity of the disease [11,12], whereas CD24 knockout mice were resistant to EAE induction [12]. The role of CD24 was also examined in a mouse model of Parkinson’s disease [13]. Although it did not directly affect neuroprotection, CD24 was necessary to mediate the neuroprotective effects of glial cell-derived neurotrophic factor (GDNF).

Furthermore, in a recent study using CD24 knockout mice, we observed that CD24 is an essential player in inflammatory bowel disease pathogenesis [14]. These observations were strengthened by clinical data suggesting that polymorphism of CD24 is associated with increased risk of autoimmune diseases [15] and with susceptibility to inflammatory bowel disease [16], as well as with non-alcoholic fatty liver disease [17].

The link between the immune system and the nervous system, which was acknowledged over four decades ago, is often neglected. For example, the pro-inflammatory cytokine interlukin-1 (IL-1) was first considered as an immune factor that controls the differentiation and activation of lymphocytes but in 1986 was discovered to influence the hypothalamic–pituitary–adrenal axis (HPA) axis, acting in the central nervous system (CNS) [18]. Thereafter it was soon realized that IL-1 plays a role in many CNS functions—from pain modulation [19,20,21] to learning, memory, neural plasticity, and neurogenesis [22]. This example illustrates that sometimes seemingly peripheral immune modulators have immense consequences at the whole-organism level [23]. Immune modulators are also involved in anxiety and depression [24]. Considering the importance of CD24 in the immune system, we sought to explore its role in learning and emotional regulation.

In the developing CNS, CD24 is expressed in developing postmitotic neurons and in growing axons [25], while in the adult brain, CD24 is also expressed in neurons in areas exhibiting neurogenesis [25] as well as in microglia [26]. CD24 involvement in neurogenesis may indicate its relevance to cognitive functions and neurodegeneration. However, that has not been explored yet. Thus, we aimed to investigate, for the first time, the effect of CD24 knockout on cognitive and emotional functions. The link between CD24 and various cognitive and emotional functions could lead to a new diagnostic or therapeutic tool for neurogenerative or mood disorders.

Although, as reviewed above, CD24 knockout or inhibition has been used to test various biochemical mechanisms and neurological conditions, to the best of our knowledge, the association between CD24 and behavioral phenotypes has not yet been examined. Our study aims to characterize the anxiety-like behavior, depression-like behavior, and cognitive performance of CD24 knockout mice compared with CD24 wild-type (WT) mice. To pursue this aim, we characterized the behavior of the mice and their cognitive performance at three time-points: adolescence (2 months), young adulthood (4 months), and adulthood (6 months).

## 2. Materials and Methods

Animals—All experiments were performed on 16 male mice: 8 CD24 wild-type (WT; control mice strain C57B/6J) and 8 CD24^−/−^ knockout (KO), in 3 different cohorts at each time-point (2 months, 4 months, and 6 months). Mice were bred at the animal facility of the Tel Aviv Sourasky Medical Center, Tel Aviv, Israel. CD24^−/−^ mice were kindly provided by Prof. Peter Altevogt (German Cancer Research Center, Heidelberg, Germany). These CD24^−/−^ KO mice are genetically tested regularly by PCR analysis of DNA obtained from tail biopsies at the age of 5 weeks. The expression of CD24 has also been verified by FACS analysis on heparinized peripheral blood samples collected from the orbital sinus of mice from the same cohorts that were not used for the behavioral test. Before the behavioral evaluations, mice were transferred to the animal facility of the Open University in Jerusalem and allowed several weeks for acclimation. Mice were housed in a room with a 12 h:12 h reversed light/dark cycle and received a chow diet and water ad libitum.

### 2.1. Genotype Verification by FACS Analysis

**Figure 1:**
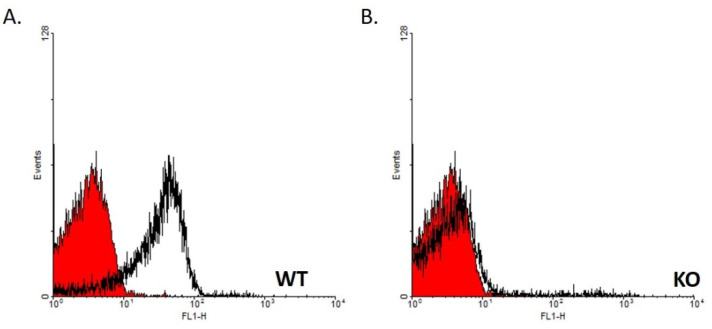
The genotype of the mice was verified by FACS analysis, based on the absence of CD24 expression on the surface of erythrocytes from the KO mice. Heparinized peripheral blood samples of WT (**A**) and KO (**B**) mice were collected and analyzed for CD24 expression. The *X*-axis (FL1-H) represents florescence magnitude (to the fluorescein isothiocyanate-conjugated goat anti-rat antibody). The red curves represent the negative control (secondary antibody only), and the black curves represent the binding of M1/69 anti-CD24 antibody.

Heparinized peripheral blood samples (100 µL) were collected from the orbital sinus of WT (Figure 1A) and KO (Figure 1B) mice and diluted in PBS. The samples were then centrifuged at 2000 rpm for 5 min. The pellet was incubated with 100 µL of rat anti-CD24 antibody, clone M1/69, at 4 °C for 30 min. Cells were washed twice in FACS buffer (10% FCS, 0.01% sodium azide in ice-cold PBS). Then, 100 µL of 1:100 diluted fluorescein isothiocyanate (FITC)-conjugated goat anti-rat antibody (Jackson Immunoresearch Laboratories, Inc 872 West Baltimore Pike, West Grove, PA, USA 19390., 112-095-003) was added to the pellet and incubated at 4 °C for 30 min. Cells were washed twice with FACS buffer, and bound antibodies were detected on a FACSCalibur™ and analyzed using CellQuestio.

### 2.2. Behavioral and Cognitive Evaluations

All the behavioral tests were done at a monitored room temperature (22–23 °C) under red-light conditions in the dark phase of the animal’s dark/light cycle in order to minimize the stress levels. Furthermore, mice were allowed to habituate to the room for 30 min before the tests. All the behavioral test were conducted at 2, 4, and 6 months of age, except for the Morris water maze (MWM), which was conducted only at 6 months.

Open Field Test (OFT)—This test reflects the conflict between the innate fear that mice have of a novel open field’s central area versus their desire to explore new environments. The test was performed as previously described by us [27]. The OFT consists of an empty square arena (40 × 40 × 40 cm) divided into 36 identical squares and surrounded by Perspex opaque walls. When anxious, the natural tendency of mice is to prefer staying close to the walls. Mice were placed in the center of the arena, and their behavior was video recorded for a total of 5 min and later coded by an observer blind to the genotype of the mice. The arena was thoroughly cleaned with ethanol and allowed to dry between subjects to eliminate any odor cues. Anxiety-like behavior was measured by the time spent in the central area (periphery was defined as 1.5 squares/10 cm from the wall). The percentage of time the animal spent moving was measured and used to assess motor functions.

Elevated plus-maze (EPM)—EPM is based on the natural tendency of mice to avoid open and elevated places and was performed as previously described by us [28]. The apparatus, situated 40 cm above the floor, consisted of a plus-maze with two black plastic closed arms and two opposite open arms. Each mouse was placed in the center of the EPM, and its behavior was video recorded for 5 min. The maze was thoroughly cleaned with ethanol and allowed to dry between subjects to eliminate any odor cues. Anxiety-like behavior was measured by the time the animals spent in the maze’s open, unprotected arm. 

Y maze (YM)—The YM assay is based on the tendency of mice to explore a new environment and was performed as previously described by us [29]. The maze consisted of three 30 cm long identical arms, 120° apart, with each arm presenting different visual cues. During the first session, the mouse was placed at the start of the distal edge of the arm with one of the two remaining arms blocked and left to explore the maze for 7 min. The mouse was then returned to its home cage and reintroduced to the start arm 5 min later for 2 min, with the two remaining arms open. The time the mouse explored the two arms was recorded, normalized, and used to calculate the preference index (PI). The preference index (PI) was calculated as the difference between the relative time (RT) for the novel and the RT for the familiar arms (RT for novel arm minus RT for familiar arm/total time exploring both arms, i.e., RT for novel arm plus RT for familiar arm). The maze was thoroughly cleaned with ethanol and allowed to dry between subjects to eliminate any odor cues.

Novel Object recognition (OR)—OR utilizes the natural tendency of mice to explore novel stimuli and was performed as previously described by us [29]. The assay consisted of two parts: a familiarization session and a test session. Twenty-four hours before the assay, the mice were allowed to explore the black Plexiglass arena (60 × 60 cm) without any objects for 5 min to get familiar with their surroundings. During the first session of the assay, the mouse was left to explore the arena for 5 min with two identical objects located 10 cm from the side walls. Twenty-four hours later, the mouse was introduced into the arena for the test session, in which one of the familiar objects was replaced with a novel object. The time spent by the mouse exploring each object was recorded for 5 min, and the relative time (RT) of exploration was calculated as the time spent exploring each object over the total exploration time for both objects. The arena was thoroughly cleaned with ethanol and allowed to dry between subjects to eliminate any odor cues.

Forced swim test (FST)—The FST was used to monitor depression-like behavior and was performed as previously described by us [30]. This paradigm is based on immobility as a measure of behavioral despair. Shortly after being placed in a transparent plexiglass cylinder (20 cm diameter) filled with water (25 °C) to a depth of 12 cm, mice were video recorded for 6 min. Total time spent immobile during the last 4 min was measured. Immobility was defined as the cessation of limb movements except for minor movements necessary to keep the mouse afloat.

Tail suspension test (TST)—Mice behavioral despair was evaluated in the TST by measuring immobility and was performed as previously described by us [30]. Mice were suspended from a horizontal bar by taping the tip of their tail to the bar for 6 min, and the time spent in immobile positions during the last 4 min was recorded.

Morris water maze (MWM)—This classical spatial learning assay was performed as previously described by us [29] using a circular pool (120 cm diameter) filled with water (23 °C) with a transparent platform (10 × 10 cm) fixed 2 cm below the surface of the water in a constant location. The pool was situated in a room containing distal visual cues, and additional cues were placed on the inner walls of the pool. The mouse was introduced into the pool at different starting points and allowed 60 s to find the platform and an additional 30 s to stay on the platform. If the mouse failed to find the platform within 60 s, it was guided to the platform and allowed to stay there for 30 s. Each mouse swam 4 times per day for 3 consecutive days. The time the mouse took to find the platform was recorded. On the 4th day of the assay, the platform was removed, and the mouse was allowed to swim for 60 s (“probe test”). The time spent in the correct quadrant (the location of the missing platform) was recorded.

All the behavioral assays were recorded and analyzed using the Biobserve software.

### 2.3. Data Analysis

All results are presented as mean ± standard error of the mean. Comparisons were carried out either by a two-tailed Student *t*-test or the Mann–Whitney U test (where normality assumption was not met). The level of significance was set at *p* < 0.05.

## 3. Results

### 3.1. Anxiety-Like Behavior

**Figure 2:**
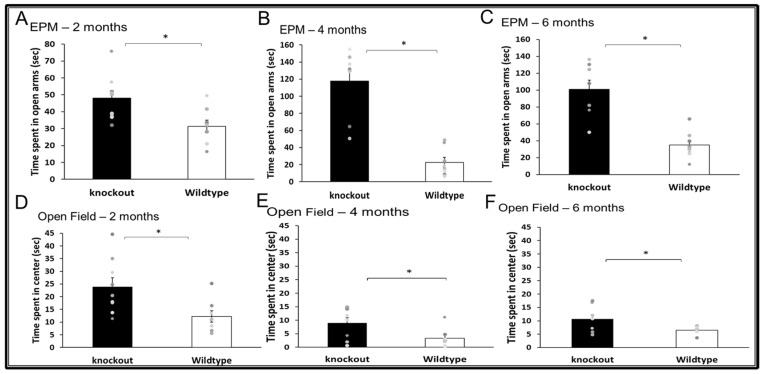
The effect of CD24 knockout on anxiety-like behaviour. Abolition of CD24 reduced anxiety-like behaviour in the EPM (**A**–**C**) and Open field test (**D**–**F**), at 2 months (**A**,**D**), 4 months (**B**,**E**) and 6 months (**C**,**F**). *n* = 8 mice per group. * indicated *p* < 0.05.

WT and CD24^−/−^ mice were assessed at the age of 2, 4, and 6 months. At the age of 2 months, CD24 knockout mice exhibited reduced anxiety-like behavior compared with age-matched WT mice in the EPM (T(15) = 2.820, *p* = 0.013; Figure 2A) and the OFT (T(15) = 2.673, *p* = 0.017; Figure 2D). Similar effects were observed at the age of 4 months in the EPM (U = 0.001, *p* < 0.001; Figure 2B) and OFT (U = 11, *p* = 0.049; Figure 2E), as well as at 6 months of age in the EPM (T(13) = 5.11, *p*= 0.000978; Figure 2C) and OFT (T(13) = 2.252, *p* = 0.042; Figure 2F).

### 3.2. Cognitive Functions

**Figure 3:**
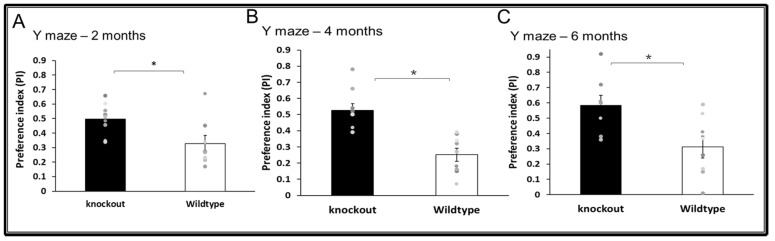
The effect of CD24 knockout on short-term memory. Abolition of CD24 increased short term memory in the Y-maze at 2 months (**A**), 4 months (**B**) and 6 months (**C**). *n* = 8 mice per group. * indicated *p* < 0.05.

WT and CD24^−/−^ mice were assessed at the age of 2, 4, and 6 months. At the age of 2 months, CD24 knockout mice exhibited improved short-term memory in the Y maze (T(15) = 2.580, *p* = 0.021) compared with age-matched WT mice (Figure 3A). Similar effects were observed at the age of 4 months in the Y maze (T(15) = 4.461, *p* = 0.000441; Figure 3B) as well as at 6 months (T(13) = 2.266, *p* = 0.02; Figure 3C). There was no difference in the total time spent in exploring both arms between the WT and KO mice, indicating that exploratory behaviours did not affect the cognitive difference between the groups (for more details see Supplementary file, Figure S3).

**Figure 4:**
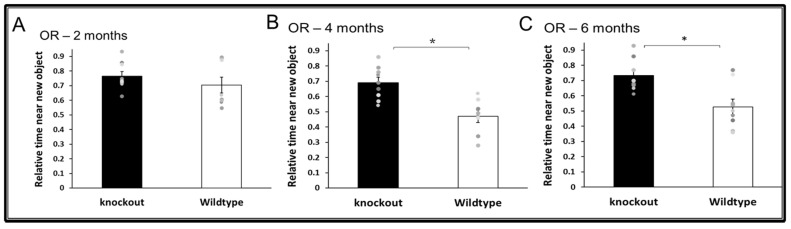
The effect of CD24 knockout on long term episodic memory. Abolition of CD24 increased long term episodic memory in the novel object recognition test at 4 months (**B**) and 6 months (**C**). No difference was observed at 2 months (**A**). *n* = 8 mice per group. * indicated *p* < 0.05.

WT and CD24^−/−^ mice were also assessed for long-term episodic memory in the novel object recognition test. At the age of 2 months, CD24 knockout mice did not exhibit improved long-term memory compared with age-matched WT mice (Figure 4A). However, significant improvement of CD24 knockout cognitive function compared with WT mice was observed at the age of 4 months (Figure 4B) and 6 months (Figure 4C). CD24^−/−^ showed higher preference of the novel object at 4 months (T(15) = 4.113, *p* = 0.001) and 6 months (T(15) = 3.266, *p* = 0.005), compared with WT mice. No difference was found in the first training season in the time spent exploring both ob-jects, indicating that exploratory behaviours did not affect the cognitive difference ob-tained in the test phase (for more details see Supplementary file, Figure S2).

**Figure 5:**
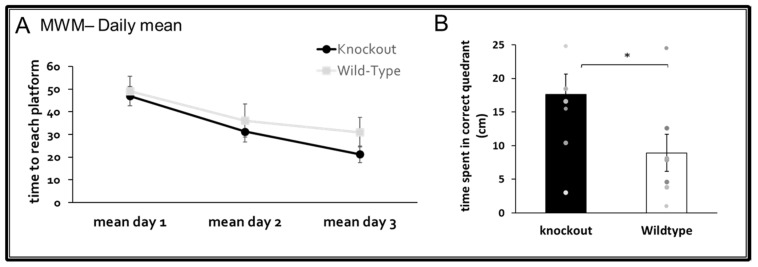
The effect of CD24 knockout on long-term spatial memory. Abolition of CD24 improved long-term memory in the radial water maze at 6 months. A significant difference was found at the probe test (**B**, * *p* < 0.05), while a trend for better spatial learning was found at the final learning stage (**A**). *n* = 8 mice per group.

In adulthood, WT and CD24^−/−^ mice were also assessed for long-term spatial memory in the Morris water maze test. At the age of 6 months, CD24 knockout mice exhibited improved spatial long-term memory in the probe-phase of the test compared with age-matched WT mice (U = 13, *p* = 0.049; Figure 5B). There was also a trend toward better learning skills of CD24 knockout mice on the 3rd day of the test. (Figure 5A). There was no difference in velocity between the WT and KO mice during any of the test phases indicating that motor functions did not influence the cognitive results (for more details see Supplementary file, Figure S1).

### 3.3. Depression-Like Behavior

**Figure 6:**
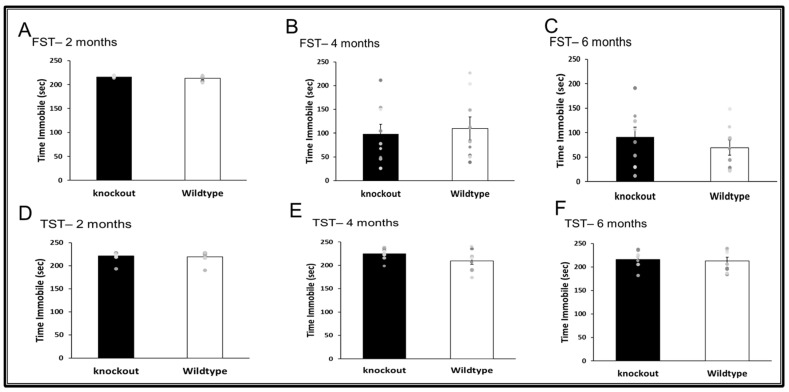
The effect of CD24 knockout on depression-like behaviour. Abolition of CD24 did not change depression-like behaviour in the FST (**A**–**C**) and TST (**D**–**F**), at 2 months (**A**,**D**), 4 months (**B**,**E**) and 6 months (**C**,**F**). *n* = 8 mice per group.

WT and CD24^−/−^ mice were assessed at the age of 2, 4, and 6 months for depression-like behavior in the FST (Figure 6A–C) and TST test (Figure 6D–F). There were no differences in depression-like behavior between WT and CD24^−/−^ mice.

### 3.4. Motor Functions

**Figure 7:**
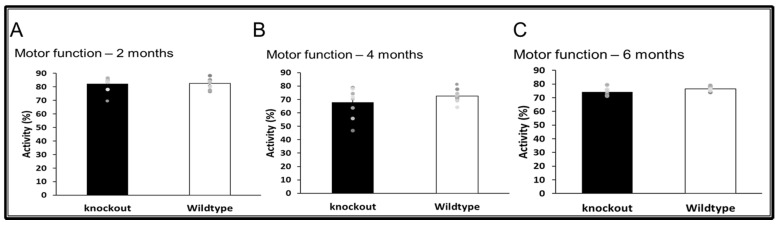
The effect of CD24 knockout on motor function. Abolition of CD24 did not effect motor function at 2 months (**A**), 4 months (**B**) and 6 months (**C**). *n* = 8 mice per group.

WT and CD24^−/−^ mice were assessed at the age of 2 (Figure 7A), 4 (Figure 7B), and 6 (Figure 7C) months. There was no difference in activity level between WT and CD24^−/−^ mice at any of the time points.

## 4. Discussion

In the present study, we report, for the first time, the behavioral phenotype of CD24^−/−^ mice. CD24^−/−^ mice exhibited better cognitive performance and less anxiety-like behavior compared with WT mice. This phenotype was constant from adolescence (2 months old) up to mature adulthood (6 months of age).

In adult mice, CD24 is involved in the development of the neuronal network. CD24 expression is maintained in adulthood in secondary neurogenesis regions, such as in the hippocampus [25], and it can promote neurite outgrowth in cerebellar neurons and hippocampal neurons [31]. The hippocampus formation is well known for its role in different types of learning and memory [32,33] and it is one of the few brain regions showing neurogenesis in adulthood [34]. This unique quality was linked both to improved cognitive function and to better emotional regulation [35]. Belvindrah et al. showed that adult mCD24^−/−^ mutant mice exhibited increased neurogenesis in the subventricular zone (SVZ) and in the dentate gyrus (DG) of the hippocampus [36]. Accordingly, we examined the mice in three hippocampus-dependent memory tests: the Y maze, which relies on short-term spatial memory [37]; the object recognition test, which relies on the consolidation of long-term episodic memory [38]; and the Morris water maze test, which relies on spatial memory [39]. The CD24^−/−^ mice had better cognitive performance in all these hippocampus-dependent memory tasks, as they spent more time in the new arm of the Y-maze, near the new object in the recognition test, and in the correct quadrant in the Morris water maze test compared with WT mice. However, our results contradict a recent report showing that the pharmacological blockade of CD24 worsened the cognitive impairment after traumatic brain injury [40]. The different pathological conditions, i.e., naïve animals vs. injured, could explain the discrepancies between that study and ours. Importantly, it was suggested that CD24 might play a dual effect in neurogenesis in response to different neuronal and immunological situations [31]. These kinds of dual effects are not uncommon in the immune system [41], and it was suggested that this quality of the innate immune system might be involved in the pathogenesis of Alzheimer’s disease (AD) [42].

In addition to CD24 effect on cognition, we also demonstrated its role in emotional regulation. As mentioned earlier, adult CD24^−/−^ mutant mice exhibited increased neurogenesis in the hippocampus [36]. It was previously shown that inhibition of neurogenesis in the hippocampus is associated with reduced anxiety but did not affect depression-like behavior [43]. These data are consistent with our results, which show that CD24^−/−^ mice had less anxiety-like behavior but did not differ in depression-like behavior compared with WT mice.

In order to exert its effect in cells, CD24 interacts with numerous associated proteins for signal transduction. These include extracellular signal-regulated kinase (ERK) 1/2 [4], p38 mitogen-activated protein kinases (p38 MAPK) [4], and nuclear factor-κB (NF-kB) [44,45]. NF-kB plays a role in inflammation and synaptic plasticity [4,45], but it is also connected to mood disorders [46]. P38 MAPK signaling pathway activation is known to be involved in various stress responses [47,48], as well as in cognitive dysfunction and synaptic plasticity [49]. In vivo, pharmacological inhibition of p38 MAPK yielded significant anti-anxiety-like activity [50], whereas in vitro, the expression of CD24 was correlated with activation of ERK1/2 and p38 MAPK [4]. Thus, in our CD24^−/−^ mice, the lack of CD24 may have lowered the p38 MAPK pathway’s activation and reduced anxiety-like behavior. 

The present results suggest that CD24 may influence important behavioral aspects at the whole-organism level. It should be noted that our study only examined male mice, and future studies should elaborate on these results and examine them in females also. Even though CD24 knockout mice are used to investigate seemingly un-related models of cognitive deficits, these impairments should not be disregarded. For example, CD24 is extensively used to investigate cancer [3], and it has been shown that cancer patients often suffer from ‘cancer-related cognitive impairments (CRCI) in memory, attention and executive functions’ [51,52]. Likewise, Parkinson’s disease, which is also investigated in relation to CD24 [11], is known to result in some cognitive decline [53].

CD24 is emerging as a potential marker for personalized treatments of cancer, and we previously showed the beneficial effect of CD24 antigen-based treatment in a cancer model [7]. Our present results suggest that CD24 might have a similar role in neurogenerative diseases.

The communication between the immune system and the CNS is well documented [54]. Chronic neuroinflammation is thought to play a role in the pathophysiology of AD [55], and this line of investigation opens new treatment venues in various inflammatory and neurodegenerative diseases [23]. The involvement of CD24 in cognitive performance and neurogenesis might suggest that CD24 plays a role in neurodegeneration, which may make this model useful in examining neurodegenerative diseases, such as AD. Therefore, it can be suggested that CD24 may alleviate the symptoms related to the disease. Hence, a potential new therapy that can halt, revert, or even prevent AD and other neurodegenerative diseases could be based on the downregulation of CD24, for example, by the administration of antibodies targeting CD24. There is no doubt that this is only the first step in a long road, but as currently there is no treatment for AD, the possibility of using such a method in neurodegeneration is intriguing and should be explored in future research. 

To conclude, this work is the first to examine the cognitive and emotional characterization in healthy CD24 knockout mice. Our results suggest that downregulation of CD24 may improve memory and anxiety-like behavior. Together with previous reports, these data emphasize the importance of CD24 to cognitive performance and anxiety and should be taken into consideration when using this model, which may lay the path for new therapies.

## Supplementary Materials

The following are available online at https://www.mdpi.com/2075-4426/11/2/105/s1, Figure S1: CD24^-/-^ and WT mice exhibited no difference in velocity in the Morris water maze test, indicating no exploratory/anxiety factors influence on the cognitive results, Figure S2: The effect of CD24 knockout on exploration during the familiarization phase. Abolition of CD24 did not effect exploration of same objects at childhood (2 months; A), adolescence (4 months; B) and auulthood (6 months; C), Figure S3: The effect of CD24 knockout on exploration. Abolition of CD24 did not effect total exploration time at childhood (2 months; A), adolescence (4 months; B) and auulthood (6 months; C).
