# Supplementary material for: Behavioral Characterizing of CD24 Knockout Mouse—Cognitive and Emotional Alternations
Source: J Pers Med. 2021 Feb 6;11(2):105. doi: 10.3390/jpm11020105 (PMC7915412; doi:10.3390/jpm11020105)
Supplement: Supplementary file 1 [file jpm-11-00105-s001.pdf]

Supplementary file 1 – analysis of motor and exploratory/anxiety factors in the cognitive test

All the tests were done in a monitored room temperature (22-23 Celsius) under red-light conditions in the dark phase of the animals dark/light cycle, in order to minimize the stress levels. Furthermore, mice were allowed to habituate to the room for 30 minutes before the tests.

Morris water maze – This method has been used for decades as a reliable method evaluate hippocampus-dependent spatial and cognitive functions. In order to assure that no other factors contributed to cognitive difference between the groups in our study, we examined the velocity (cm/c) of the mice. There was no difference in velocity between the WT and KO mice during the 3 training seasons (Fig1.A) and probe-test (Fig1B), indicating that motor or exploratory/anxiety factors did not influence the cognitive results.

Figure 1

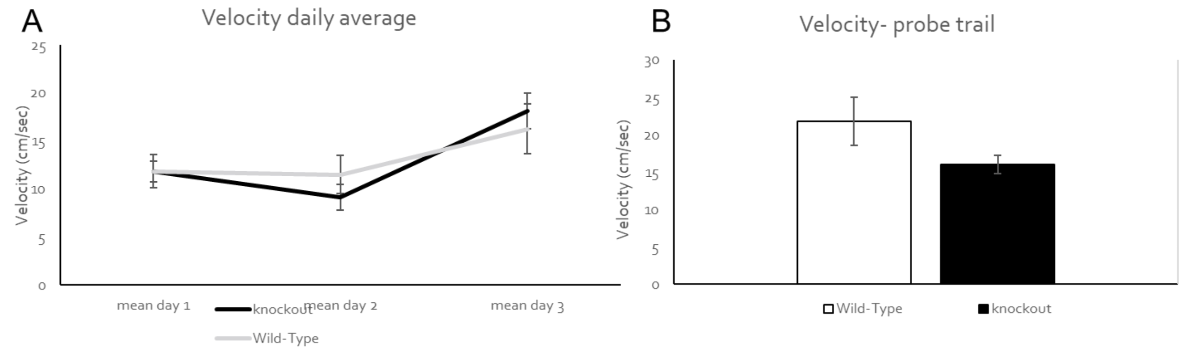

Figure 1 – CD24$^{-/-}$ and WT mice exhibited no difference in velocity in the Morris water maze test, indicating no exploratory/anxiety factors influence on the cognitive results.

The Novel Object Recognition test – The NOR is a well-known and reliable test for memory and learning that has been in use for more than 20 years. It yielded consistent and reliable results in a wide variety of fields and strains. The protocol of the test included 3 phases done in 3 consecutive days – habituation phase to the arena (which is the open field test reported in the main article), familiarization phase and test phase. As mentioned in the main article, no difference was found in the distance walked between the two groups on the habituation phase of the test. We also calculate the time spent near the similar objects in the familiarization phase in order to ensure that exploratory or anxiety-related behaviours did not affect the cognitive difference obtained in the test trail. In the age of 2 (Fig 2A) ,4 (Fig 2B) and 6 (Fig 2C) months there was no difference in the first training season in the time spent exploring both objects, indicating that exploratory/anxiety-related behaviours did not affect the cognitive difference obtained in the test phase.

Figure 2

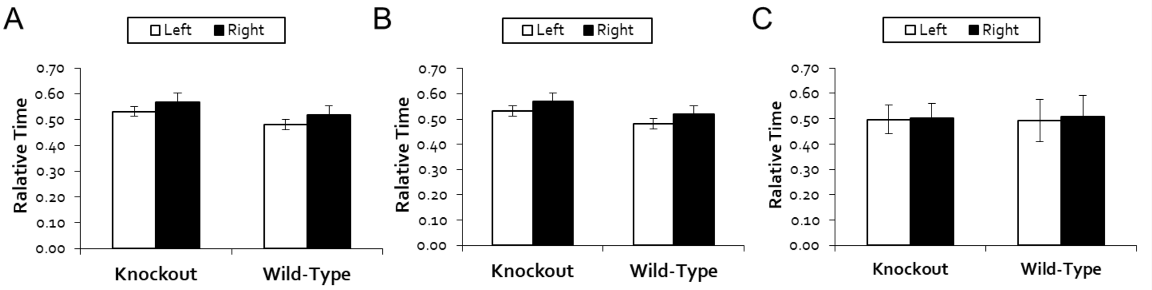

Figure 2 – The effect of CD24 knockout on exploration during the familiarization phase
Abolition of CD24 did not effect exploration of same objects at childhood (2 months; A), adolescence (4 months; B) and adulthood (6 months, C).

Forced choice Y-maze –The Y-maze is a non-aversive task consistent from a Y-shaped maze with high walls. It is a well-documented and reliable method for the assessment of short-term memory. In the age of 2 (Fig 3A) ,4 (Fig 3B) and 6 (Fig 3C) months there was no difference in the total time spent in exploring both arms between the WT and KO mice, indicating that exploratory/anxiety-related behaviours did not affect the cognitive difference between the groups.

<u>Figure 3</u>

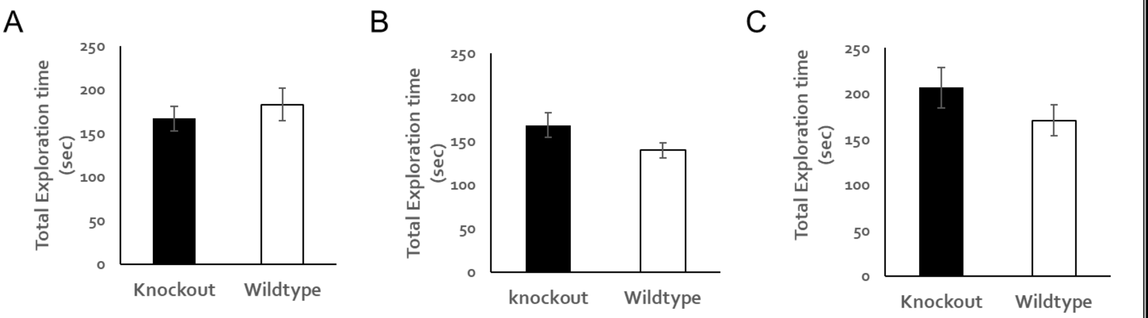

Figure 3 – The effect of CD24 knockout on exploration
Abolition of CD24 did not effect total exploration time at childhood (2 months; A), adolescence (4 months; B) and adulthood (6 months, C).

In Summary, in all the cognitive test there was no difference in exploratory/anxiety behaviour or motor functions that may underline the performance differences observed between the CD24-Knockour and the WT mice.
